# Deformable registration of 3D ultrasound volumes using automatic landmark generation

**DOI:** 10.1371/journal.pone.0213004

**Published:** 2019-03-15

**Authors:** Michael Figl, Rainer Hoffmann, Marcus Kaar, Johann Hummel

**Affiliations:** Center for Medical Physics and Biomedical Engineering, Medical University of Vienna, Vienna, Austria; North Shore Long Island Jewish Health System, UNITED STATES

## Abstract

US image registration is an important task e.g. in Computer Aided Surgery. Due to tissue deformation occurring between pre-operative and interventional images often deformable registration is necessary. We present a registration method focused on surface structures (i.e. saliencies) of soft tissues like organ capsules or vessels. The main concept follows the idea of representative landmarks (so called *leading points*). These landmarks represent saliencies in each image in a certain region of interest. The determination of deformation was based on a geometric model assuming that saliencies could locally be described by planes. These planes were calculated from the landmarks using two dimensional linear regression. Once corresponding regions in both images were found, a displacement vector field representing the local deformation was computed. Finally, the deformed image was warped to match the pre-operative image. For error calculation we used a phantom representing the urinary bladder and the prostate. The phantom could be deformed to mimic tissue deformation. Error calculation was done using corresponding landmarks in both images. The resulting target registration error of this procedure amounted to 1.63 *mm*. With respect to patient data a full deformable registration was performed on two 3D-US images of the abdomen. The resulting mean distance error was 2.10 ± 0.66 *mm* compared to an error of 2.75 ± 0.57 *mm* from a simple rigid registration. A two-sided paired t-test showed a p-value < 0.001. We conclude that the method improves the results of the rigid registration considerably. Provided an appropriate choice of the filter there are many possible fields of applications.

## Introduction

Ultrasound imaging (US) is real-time, non-invasive and less expensive than many other clinical imaging methods. However, the images are often noisy, contain several typical artefacts and are affected by soft-tissue deformation caused by the application of the US-scan itself. All these factors can contribute to difficulties in the comparison of images of the same object at different times or from different positions i.e. the registration of US images [1]. In order to improve monitoring and diagnostics using standard US technologies there is a certain demand for finding better ways to compare and fuse corresponding images.

Deformable image registration is applied additionally to rigid image registration techniques as described in [1–3]. Within the last few years, different approaches for deformable image registration were established. It is assumed that deformations are elastic movements of tissue caused by external forces, weight displacements or muscular activity. All models have therefore to be applied in a way that the resulting displacement field represents a situation that is physiologically plausible and must preserve tissue topology. An overview of recent methods for US registration including many deformable approaches can be found in [1].

Three main approaches can be distinguished: geometric models derived from physical models, geometric models derived from interpolation and knowledge based transformations. There are physical models using the theory of elasticity [4], viscosity [5] or diffusion [6].

In interpolation theory deformation is assumed to be known exactly and assigned to fiducial data. Images are deformed according to these fiducial data depending on their distance. For regions far away the influence of the fiducial data points vanishes. Among a large family of interpolation methods, elastic splines, radial basis functions and piecewise affine models should be mentioned. Elastic Body Splines were introduced by Davis et al. [7]. Splines represent a local deformation with well defined differentiable connection conditions to neighboring elements [8]. Apart from the preservation of topology these methods require defined forces which sufficiently represent reality. Kohlrausch et al. [8] assumed that forces decrease with their distance to landmarks according to Gaussian functions. Yang et al. [9] applied parametrized mathematical functions (radial base functions) to describe deformation. Piecewise affine models extend rigid registration techniques by assigning affine transformations to local points [10, 11]. Deformations are given by adapting the parameters of the affine transformation by a local linearisation.

The method used in the present work can be seen a mixture of different geometric models [12]. The calculation of the deformation field is based on landmarks that describe saliencies like organ boundaries or vessels using plane fits to previously defined structures. In contrast to spline based techniques, planes are locally assigned to linearise the organ surface without any boundary condition to the neighborhood. Since the focus is set on surfaces, the method is knowledge based as well and only suitable for tissue regions where saliencies can be found. However, this limitation is compensated by the simplicity of the method since the calculation is straight forward and non iterative.

We evaluated our registration method based on [12] using a US phantom and an abdominal 3D-US volume of a volunteer. A detailed analysis of the target registration error (TRE) was developed.

## Materials and methods

**Fig 1:**
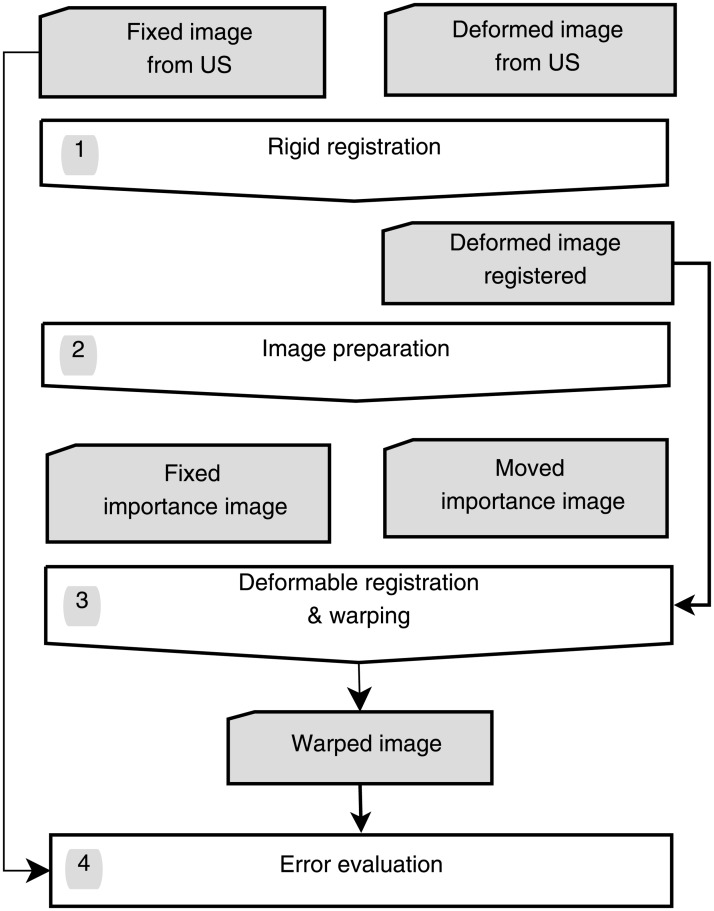
Work flow showing the deformable registration process and the error evaluation.

The work flow of the complete registration process including the calculation of the TRE is shown in Fig 1. It was divided into four main parts.

*First*, a *rigid registration* is applied to avoid large offsets which could not be corrected by the deformable registration only.

*Second*, the US images were preprocessed to emphasize important structures such as edges and corners (see the appropriate subsection on *Image preparation*). These structures were then used for the subsequent deformable registration.

*Third*, the deformable registration was applied. For this step saliencies in the images were used. It was assumed that the images contained structures that could clearly be identified as surfaces (e.g. vessels and organs such as the urinary bladder).

*Finally*, the whole registration was quantified by calculating the TRE before and after the warping process. If successful, the TRE is expected to be considerably smaller than before.

### Rigid registration

The metrics implemented were normalized cross correlation (NCC) and mutual information (MI). The latter showed better results and was used for all rigid registrations subsequently [3]. We used a multi-level optimizer which sampled down the input volumes to coarser resolutions for the early stages of the registration. The Insight Segmentation and Registration Toolkit ITK (Kitware, Inc. NY, USA) was used for registration and Qt4 (The Qt Company, Finland) for the user interface.

### Image preparation—Importance images

Image preprocessing followed a method described in [12] where the *importance images* were generated. In the following we denoted the pre-operative image as fixed image *I*_*f*_(*x*, *y*, *z*) and the interventional image as deformed image *I*_*d*_. First, a gradient filter was applied to emphasize edges and define surfaces. This resulted in gradient images *grad*_*f*,*d*_. In a next step, corners were emphasized. Corners correspond to the second derivative, therefore a Laplacian operator was applied. Since taking the derivative increases noise, a Gaussian smoothing filter was applied before the Laplacian. The resulting images were stored as Laplacian (of Gaussian) images *LoG*_*f*,*d*_.

After this step three values were assigned to every voxel: the gray value itself, the gradient at the voxel location and the Laplacian of Gaussian. The *importance images* *Imp*_*f*,*d*_ were built up as a weighted sum:

$$\begin{array}{c} Imp_{f,d}=I_{f,d}\;w_{gray}+grad_{f,d}\;w_{grad}+LoG_{f,d}\;w_{LoG} \end{array}$$ (1)

The scalars *w*_*i*_ are weights that control the contributions of grey values and derivatives of the importance images. The image preparation was completed by a windowing process. This last step suppressed minor image details and emphasized saliencies.

### Deformable registration and warping

**Fig 2:**
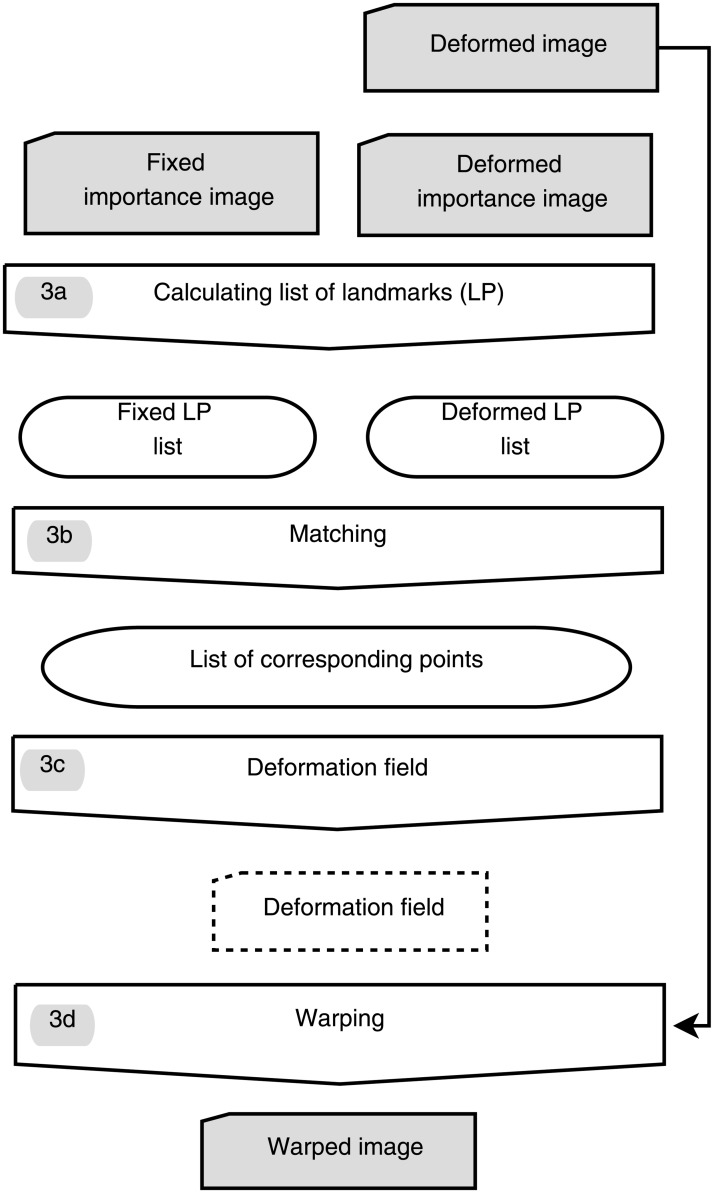
Detailed work flow for step 3. *(3a)* calculation of leading points (LPs) for each image. *(3b)* finding corresponding LP and create a list of corresponding points. *(3c)* calculation of a deformation field i.e. displacement vectors for each voxel. *(3d)* warping the deformed image.

Fig 2 shows the process for correction of deformations which corresponds to step 3 in Fig 1. This process can be divided into four steps:

#### (a) Leading points—Landmarks

Representative landmarks, the *leading points* (LPs), representing boundaries in the image were found in the importance images by thresholding (step 3a in Fig 2).

If the voxel value at a certain position *P*_*LP*_ = *P*(*x*, *y*, *z*) exceeded a certain threshold the point was considered to be representative and marked as LP, provided no further LP existed in a certain neighborhood. *P*_*LP*_ was marked as leading point if |*P*_*LP*_ − *LP*| > *ε* ∀*LP*, with *LP* a leading point which had previously been set. This restriction avoided that a region fulfilling the threshold conditions was filled with an excessive number of *LP*s. Therefore, the threshold parameter defined where the *LP*s were set and *ε* defined the density of leading points.

This was done for the fixed and the deformed image and the resulting two point sets were used to calculate the deformation.

#### (b) Matching

For the calculation of the deformation it was assumed that larger (rigid) translations and rotations have been already compensated by the rigid registration and that emerging curvatures were small.

**Fig 3:**
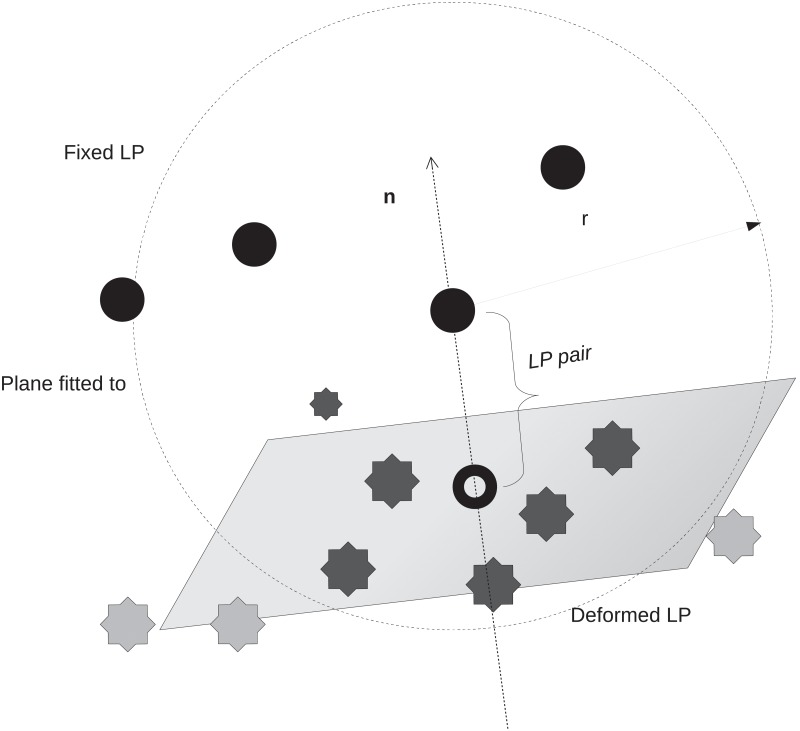
Matching process. A plane was fitted to leading points of the deformed image (dark asterisks) in a certain neighborhood to a leading point in the fixed image (circles). The new corresponding leading point *LP*_*corr*_ was the point on the plane closest to the orthogonal projection point (dough nut) in the fixed image.

The matching process is illustrated in Fig 3. Each *LP*_*fix*_ in the fixed image was assigned to a *LP*_*def*_ in the deformed image. To find the corresponding *LP*_*def*_ for a given *LP*_*fix*_ only a spherical neighborhood *ε* with radius *r* and center *LP*_*fix*_ was taken into account. In Fig 3 the candidate *LP*_*def*_ were illustrated as dark asterisks and were located on a boundary surface. As only small curvatures were assumed this surface could locally be fitted by a plane. The normal vector **n** of this plane was also indicated in the sketch. The corresponding *LP*_*corr*_ was then determined as that *LP*_*def*_ on the plane which was closest to the orthogonal projection point (shown as a doughnut in Fig 3).

*LP*_*fix*_ and *LP*_*corr*_ defined the displacement vector representing the local deformation at this point. This procedure was performed for all *LP*_*fix*_s which finally resulted in a set of displacement vectors $\;v_{n}^{{}^{LP}}≔{\vec{v}}^{{}^{LP}}({LP}_{fix,n})$.

#### (c) Deformation field

To calculate the deformation field a displacement vector $\;v_{i}=\vec{v}(x_{i})$ had to be derived for each voxel **x**_*def*,*i*_ = (*x*_*i*_, *y*_*i*_, *z*_*i*_) in the deformation image from the vectors assigned to the LPs. As deformation of tissue in a certain region was considered as an elastic response to a force, the deformation field had to be continuously differentiable. Moreover, the local movements (i.e. |**v**_*i*_|) were restricted by physiological constrains. In the present approach, deformation was derived from the movement of saliencies. Therefore, the local displacements **v**_*i*_ had to fulfill the following conditions:

- Voxels **x**_*i*_ in the neighborhood of the boundaries were deformed similar to the boundary itself. Moving vectors close to a leading point had to be similar to the moving vector of the LP itself: $|v_{i}|\approx |v_{n}^{{}^{LP}}|$.
- The influence of a LP should decrease with the distance to the voxel.
- The influence of the LPs should tend to zero for voxels that are located outside of the deformed region.
- The influence of each $v_{n}^{{}^{LP}}$ to a **v**_*i*_ should be proportional to a summation of the influences of each LP pair.

This was accomplished by calculating the local displacements as:

$$\begin{array}{c} v_{i}=\frac{1}{N}\sum_{n=1}^{N}\alpha \;e^{-|x_{n}^{{}^{LP}}-x_{i}|/\sigma^{2}}v_{n}^{{}^{LP}} \end{array}$$ (2)

The parameters *α* and *σ* allowed to control the weighting. *α* denotes an absolute value for the strength of the displacement and *σ* controls the range of displacement i.e. the region of interest (ROI).

#### (d) Warping

Given the complete deformation field the deformed image was *warped*. The new position *x*^*w*^ of each voxel was calculated by

$$\begin{array}{c} x_{i}^{w}=x_{i}+v_{i} \end{array}$$ (3)

The warping process was a reverse mapping process: in the deformed image a new gray value was calculated for every voxel. If a voxel position **x**_*i*_ was the destination of more than one moved voxel the new gray value was chosen to be the gray value of the closest LP $\left(x^{w}=min\;|x_{k}^{w}-x_{i}|\right)$.

### Error calculation

**Fig 4:**
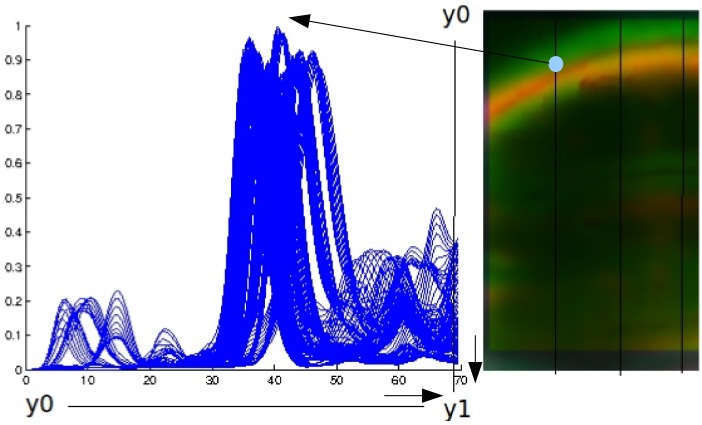
Calculation of the TRE. Defining lines *y*_0_ to *y*_1_ through an image slice (*right*) provided a function of gray values along the line (*left*). Intersection points (pale blue) with the structure were determined by finding the maximum of the function.

For calculation of the TRE representative points found on the surface (or saliences, respectively) were used. To find corresponding points in all, the fixed, deformed and warped images, lines were defined [13]. In short, the salience was displayed on each slice as an intersection curve. Lines were introduced parallel to the y-axis in a defined but constant distance. Along these lines an intensity profile could be recorded (see Fig 4). If the surface to be analyzed was the brightest object in the observed ROI, the intersection point between line and curve defined the target point.

This was done for all three images (fixed, deformed and warped) and resulted in three point sets: $\left\{S_{i}^{{}^{f}}\right\},\left\{S_{i}^{{}^{d}}\right\},\left\{S_{i}^{{}^{w}}\right\}$. The index *i* identifies corresponding points. If the error was found to be zero (i.e. the warping was perfect), the corresponding points in the fixed and the warped image were located at the same position. The total error which was assumed to be the TRE was calculated as:

$$\begin{array}{c} TRE_{f\to w}=\sqrt{\frac{1}{N}\sum_{i=1}^{N}{\left(S_{i}^{{}^{f}}-S_{i}^{{}^{w}}\right)}^{2}} \end{array}$$ (4)

This formula was also applied to the fixed and to the deformed point sets by replacing $S_{i}^{{}^{w}}$ by $S_{i}^{{}^{d}}$ in Eq 4 which defined the *TRE*_*f*→*d*_ (i.e. the error of the rigid registration without warping).

Additionally, we calculated a mean distances error (*Err*^*md*^) and the corresponding standard deviation between fixed and deformed $\left(Err_{f\to d}^{md}\right)$ and fixed and warped images $\left(Err_{f\to w}^{md}\right)$ by averaging the Euclidean distances between the fiducials. To test for significant improvement between simple rigid registration and warping process a (two sided) t-test was applied.

### Phantom experiments

**Fig 5:**
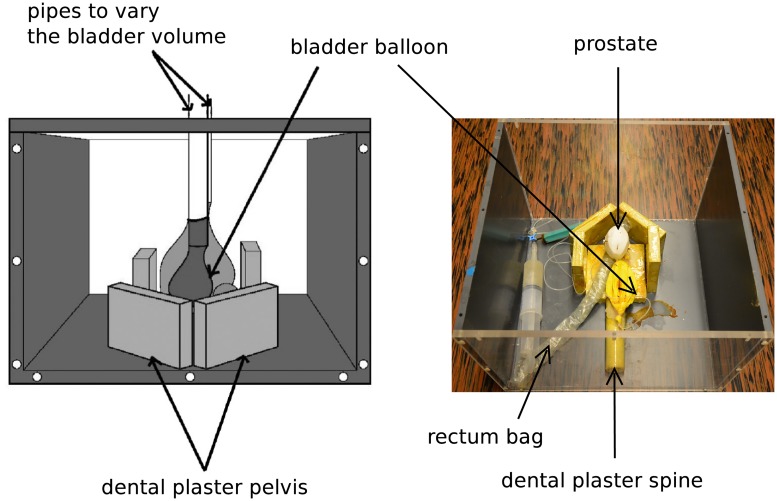
The image shows a sketch and a photo of the prostate/abdominal phantom used in the evaluation. The balloon representing the bladder can be changed in size. The tank was filled with a 5% ethanol solution to adjust the speed of sound to human tissue.

The complete warping process was evaluated using a phantom that represented the lower abdominal area and mimicked the urinary bladder and the prostate in a plastic tank (Fig 5). This phantom has already been used in [14]. The bladder was represented by an inflatable balloon. A prostate-shaped polystyrene object of approximately 110 cc was glued to the bladder balloon. The bladder was chosen to be the target structure (ROI) as its boundaries showed a clearly identifiable salience. The tank was filled with water mixed with ethanol (5% solution) to obtain the speed of sound equal to human tissue.

### Patient evaluation

With respect to patient data abdominal 3D-US images were taken. A blood vessel therein was used as the target saliency. Slight deformations of the target were caused by varying the contact pressure of the transducer.

All images were taken with a 3D-US device (General Electrics Volusion E8) and a 4MHz 3D transducer. The 3D-US images had a resolution of 255 x 255 x 120 voxels with a pixel spacing of 0.375 mm/voxel in all directions.

## Results

### Phantom data

**Fig 6:**
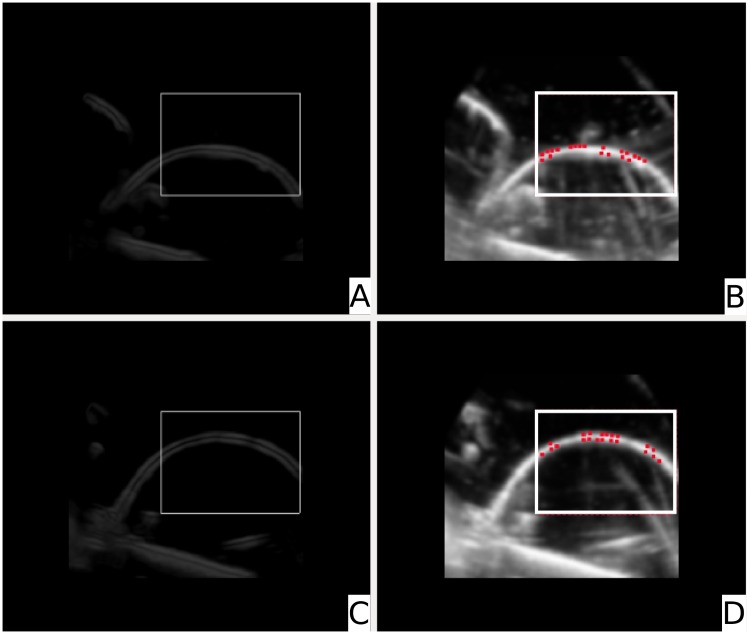
Image preprocessing and landmark calculation (phantom data). *Image A*: The windowed importance image of the fixed image. *Image B*: The fixed image with leading points assigned. *Image C*: The windowed importance image of the deformed image. *Image D*: The deformed image with LPs assigned.

The image preprocessing resulted in importance images of the fixed and the deformed image as seen in the upper and lower left images in Fig 6. Images A and C of this Fig 6 show appropriately windowed importance images of the fixed and the deformed image of the US phantom. In images B and D the LPs are displayed with red dots. As can be seen, the LPs are concentrated on the most dominant structure of the image which in this case is the balloon’s surface.

The parameters for the importance image according to Eq 4 were set to *w*_*gray*_ = 0.51, *w*_*grad*_ = 0.40 and *w*_*LoG*_ = 0.51. The windowing was carried out with a lower threshold of 10% and a upper threshold of 91%. For the calculation of the LPs a threshold of 50% and an excluding radius of *ε* = 4.18 *mm* were applied which resulted in a satisfying density of LPs. In the plane fitting process the radius was set to *r* = 5.38 *mm*. The Gaussian weights were chosen to be *α* = 150, *σ* = 2.5 (see Eq 2).

**Fig 7:**
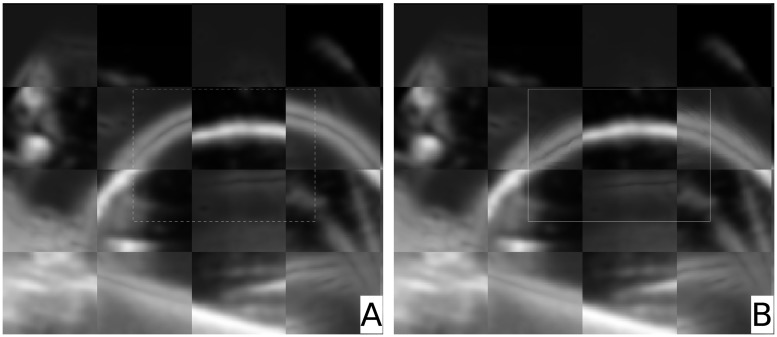
Deformable registration with phantom data. *Image A*: Checkerboard of the fixed and the deformed image after rigid registration. *Image B*: Checkerboard of the fixed and the warped image. The effect of warping (i.e. the correction of the deformation) can clearly be seen.

The result of the deformable registration for the phantom is shown in Fig 7. For a visual judgment of the registration quality we used checkerboard images of the structure where most of the LPs were found (Fig 6). Image A in Fig 6 shows a checkerboard image of the fixed and the deformed image after the rigid registration. On the right hand side, image B in Fig 6, the additional warping process was applied to the deformed image. Compared to simple rigid registration the balloon’s surface is now properly registered. The TRE according to Eq 4 calculated using 150 automatically selected points (see Fig 4) was *TRE*_*f*→*d*_ = 5.79 *mm* for the simple rigid registration and *TRE*_*f*→*w*_ = 2.02 *mm* for the warping.

The mean errors were computed to $Err_{f\to d}^{md}=5.69\pm 1.08\;mm$ and $Err_{f\to w}^{md}=1.77\pm 0.97\;mm$. A two-sided paired t-test showed a p-value < 0.001. The data can be found in the supporting information section S1 Dataset.

### Patient data

**Fig 8:**
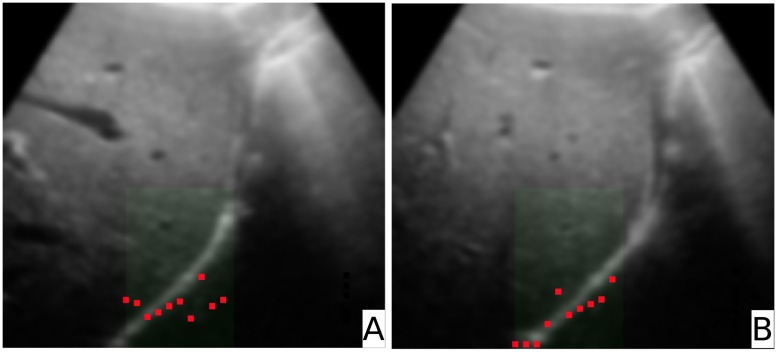
Leading points. Image **A** shows the leading points in the fixed image, image **B** shows the leading points in the moving image. These points were used for the warping process.

For the patient data a Canny edge filter was applied prior to the creation of the importance images. In Fig 8 the resulting LPs on the patient image including the blood vessel are shown. Like with the phantom data (Fig 6), the LPs are found on the most prominent structure of the image.

**Fig 9:**
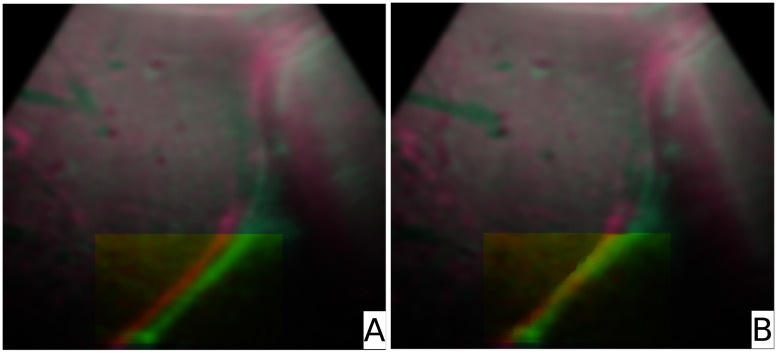
Deformable registration with patient data. The fixed image (red) is overlaid with the deformed image (green). Image **A** shows an overlay before, image **B** after the warping.

Fig 9 shows the result of the warping process with patient data. The fixed image is painted red, the deformed image green. On the left hand side (marked with ‘A’) an overlay of the fixed and deformed image after rigid registration is displayed. On the right hand side (denoted as ‘B’) the merged fixed and warped images are shown. The TREs were calculated from 107 fiducials (intersection points between lines and the structures marked manually, as defined in section ‘Error calculation’) and amounted to *TRE*_*f*→*d*_ = 2.82 *mm* and *TRE*_*f*→*w*_ = 2.21 *mm*. The mean distance errors were found to be $Err_{f\to d}^{md}=2.75\pm 0.57\;mm$ and $Err_{f\to w}^{md}=2.10\pm 0.66\;mm$. The p-value for the two sided t-test was < 0.001. The data can be found in the supporting information section S2 Dataset.

## Discussion

Our multi-resolution approach has shown significant improvements of the TRE in both evaluations, the phantom and the patient study. Furthermore, the errors found were in good accordance with [12] where registrations between liver images were performed. Due to the higher contrast in some parts of the images, we were able to evaluate more target points compared to [12]. In [15], the accuracy and variability of rigid and non-rigid registrations of transrectal 3D-US images of the prostate were evaluated. Different surface- and intensity-based rigid and nonrigid registration algorithms based on thin-plate splines and B-splines were evaluated. The pre-registration TRE was 7.36 ± 4.17 *mm* compared to a TRE of 1.96 ± 0.85 *mm* after non-rigid registration. Nevertheless, no significant difference between rigid and non-rigid registration was found. In contrast, De Silvas et al. [16] were able to reduce the TRE by 4.75 *mm* compensating prostate motion induced by the biopsy procedure. Their intensity-based 2D-3D rigid registration algorithm optimized the normalized cross-correlation metric using Powell’s method. Rivas et al. [17] applied a non-rigid US registration on brain images where a deformation occurred due to a resection of brain tumors. The deformation was modeled with free-form cubic B-splines. Their registration algorithm reduced the mean TRE from an initial value of 3.7 *mm* to 1.5 *mm*. A similar approach related to the presented registration method is given by the determination of geometric moment invariants for each point in the image [18, 19] where attribute vectors are determined from local spatial intensity histograms. Their method was applied to magnetic resonance (MR) images and has proven to be robust and reliable. The transfer of this method to US images would be challenging due to the huge amount of speckle artifacts.

In previous studies we have already applied 3D-3D US registrations on prostate images. In [3], a rigid 3D-US/3D-US registration was used for patient alignment in tele-therapy. Although the resulting TRE was acceptably small, the development of a deformable registration has already been suggested there. In a follow-up study [14], three different deformable registration methods were compared using cone beam CT image data. Although deformable registration methods improved the outcome over a rigid registration for lung cases and in a phantom study, no significant improvement was found for the prostate study.

We suppose potential applications in US guided biopsies, ethanol injection therapies and radiofrequency ablations [20] of the liver [21]. Intra-operative 3D US imaging can provide information for real-time update of tool positions, instruments or catheters during an intervention. As liver metastases are often not visible in US images [22], several attempts exist to display or merge a US image with its corresponding high-quality CT (or MR) volume. Lange et al. [23] used a vessel-based non-rigid registration for their 3D ultrasound guidance in liver surgery where segmented vessel centerlines were used.

In radiotherapy, the correct alignment of the patient is an important requirement for treatment success. One reliable and radiation-free method of capturing images of soft tissue (e.g. for prostate irradiation) applicable for patient positioning involves 3D ultrasound [3, 24, 25]. For patient alignment, a 3D ultrasound image is taken before each treatment fraction and then registered with a pre-interventional US image taken at the planning CT site. Although such systems have shown successful clinical applications, tissue deformation is still an obstacle with respect to accuracy and should be considered in the positioning process. As the prostate as well as the urinary bladder exhibits well distinguishable tissue surfaces, our method would be applicable considering appropriate filtering.

### Limitations

The novelty of the method lies in the combination of calculating landmarks and the way the saliencies are matched. As it turned out this method provides good functionality for simple saliencies. It is capable for the correction of deformation for certain regions e.g. regions that clearly provide surface structures. These conditions are given when vessels or the urinary bladder are imaged [3, 26–28]. In this sense, our approach is limited to topologically simple high contrast surfaces. Further work will focus on the extension to more complex structures, for instance tissue surfaces with large local curvatures and a complex topology. For this extension non linear regression using polynomial surfaces instead of planes had to be introduced.

Alternatively, appropriate structures such as blood vessels nearby the regions of interest could be chosen, provided the target region shows a movement similar to these vessels. As such vessels are found in various regions of the body, the variety of possible applications ranges from laparoscopy in the abdomen [29] to optical coherence tomography (OCT) imaging of human skin [30] and human eye [31]. In these cases, the LPs define surfaces which allow for fitting local planes with low TREs. Consequently, other structures can be chosen for this procedure if their saliencies are prepared by appropriate filters before the LP determination is started. In our experiments, the Canny edge filter revealed to be an excellent choice for blood vessels. Therefore, an intensive evaluation of filters for a given—or desired—structure to be registered has to be carried out in future applications. Another parameter depending on the structures in the image and considerably effecting registration quality, is the threshold of the importance image. This value changes the number of LPs and consequently also influences registration time [12].

Another limitation of our approach are shadow artifacts in US images. In such images LPs can not be created and it is not possible to define a reliable deformation within the shadowed regions. The deformation field has to be estimated from the adjacent LPs. Therefore, the reliability of such an estimation depends on the distance of the shadow regions to the next reliable LPs.

### Conclusion

We found the method to work reliable and improve the registration accuracy significantly for both the phantom and the patient images. As discussed above there are many possible fields of applications where the method can be used, provided an appropriate choice of the filter.

## Supporting information

S1 Dataset This file contains the 150 automatically selected points from the phantom that were used to calculate the TRE for the phantom registration. (CSV)

S2 Dataset This file contains 107 fiducial points from the patient data that were used to calculate the TRE for the patient registration. (XLS)
